# Machine learning-based prediction of acute severity in infants hospitalized for bronchiolitis: a multicenter prospective study

**DOI:** 10.1038/s41598-020-67629-8

**Published:** 2020-07-03

**Authors:** Yoshihiko Raita, Carlos A. Camargo, Charles G. Macias, Jonathan M. Mansbach, Pedro A. Piedra, Stephen C. Porter, Stephen J. Teach, Kohei Hasegawa

**Affiliations:** 1 Department of Emergency Medicine, Massachusetts General Hospital, Harvard Medical School, 125 Nashua Street, Suite 920, Boston, MA 02114-1101 USA; 2 Department of Pediatric Emergency Medicine, Rainbow Babies and Children’s Hospital, Cleveland, OH USA; 3 Department of Medicine, Boston Children’s Hospital, Harvard Medical School, Boston, MA USA; 4 Departments of Molecular Virology and Microbiology and Pediatrics, Baylor College of Medicine, Houston, TX USA; 5 Department of Pediatrics, College of Medicine, University of Cincinnati, Cincinnati, OH USA; 6 Division of Emergency Medicine, Cincinnati Children’s Hospital Medical Center, Cincinnati, OH USA; 7 Division of Emergency Medicine and Department of Pediatrics, Children’s National Health System, Washington, DC USA

**Keywords:** Diseases, Health care, Medical research, Signs and symptoms

## Abstract

We aimed to develop machine learning models to accurately predict bronchiolitis severity, and to compare their predictive performance with a conventional scoring (reference) model. In a 17-center prospective study of infants (aged < 1 year) hospitalized for bronchiolitis, by using routinely-available pre-hospitalization data as predictors, we developed four machine learning models: Lasso regression, elastic net regression, random forest, and gradient boosted decision tree. We compared their predictive performance—e.g., area-under-the-curve (AUC), sensitivity, specificity, and net benefit (decision curves)—using a cross-validation method, with that of the reference model. The outcomes were positive pressure ventilation use and intensive treatment (admission to intensive care unit and/or positive pressure ventilation use). Of 1,016 infants, 5.4% underwent positive pressure ventilation and 16.0% had intensive treatment. For the positive pressure ventilation outcome, machine learning models outperformed reference model (e.g., AUC 0.88 [95% CI 0.84–0.93] in gradient boosted decision tree vs 0.62 [95% CI 0.53–0.70] in reference model), with higher sensitivity (0.89 [95% CI 0.80–0.96] vs. 0.62 [95% CI 0.49–0.75]) and specificity (0.77 [95% CI 0.75–0.80] vs. 0.57 [95% CI 0.54–0.60]). The machine learning models also achieved a greater net benefit over ranges of clinical thresholds. Machine learning models consistently demonstrated a superior ability to predict acute severity and achieved greater net benefit.

## Introduction

Bronchiolitis is the leading cause of infant hospitalization in the US, accounting for 107,000 infant hospitalizations each year with direct cost of 734 million US dollars^1^. Even among hospitalized infants, the severity of bronchiolitis can range from moderate severity (which requires observation and supportive therapies, such as supplemental oxygen, fluid, and nutrition) to near-fatal and fatal infections. Previous studies have identified individual risk factors for higher severity of bronchiolitis (e.g., young age, prematurity, viral etiology)^2–5^ and developed prediction scoring models (e.g., logistic regression models)^6–9^. However, identifying the subgroup of infants with bronchiolitis who require higher acuity care (e.g., positive pressure ventilation, intensive care unit [ICU] admission) remains an important challenge. The difficulty and uncertainty of predicting acute severity—and, consequently, the appropriate level of care for infants with bronchiolitis—are reflected by the well-documented variability in inpatient management across the nation^1,10–12^.

Machine learning models have gained increasing attention because of their advantages, such as the ability to incorporate high-order, nonlinear interactions between predictors and to yield more accurate and stable predictions. Indeed, recent studies have reported that the use of machine learning models provide a high predictive ability in various conditions and settings—e.g., sepsis^13,14^, asthma exacerbation^15^, emergency department (ED) triage^16,17^, and unplanned transfers to ICU^18^. Despite the clinical and research promise, no study has yet examined the utility of modern machine learning models in predicting outcomes in infants hospitalized for bronchiolitis—a large population with high morbidity and health resource use.

In this context, we aimed to develop machine learning models that accurately predict acute severity in infants hospitalized with bronchiolitis, and compare their predictive performance with that of conventional scoring approaches^6^.

## Results

During 2011–2014, 1,016 infants with bronchiolitis were enrolled into a 17-center prospective cohort study. The median age at the enrolment was 3.2 months (IQR 1.6–6.0), 40% were female, and 42% were non-Hispanic white. The length-of-hospital stay varied widely from 0 to 60 days (median, 2 days) (Table 1). Clinical data had a small proportion of missingness; most had < 1% missingness (e.g., missingness on oxygen saturation with the use of supplemental oxygen, 0.1%) while the maximum proportion of missing was 4.8% (eTable 3 in Additional file 1). Overall, 55 infants (5.4%) underwent positive pressure ventilation and 163 infants (16.0%) had intensive treatment outcome.

**Table 1:** Patient characteristics and clinical outcomes in 1,016 infants hospitalized for bronchiolitis.

Variables	n = 1,016
**Demographics**	
Age (month), median (IQR)	3.2 (1.6–6.0)
Female sex	406 (40.0)
Race/ethnicity	
Non-Hispanic white	430 (42.0)
Non-Hispanic black	239 (23.5)
Others	347 (34.2)
**Medical history**	
Prenatal maternal smoking	147 (14.7)
Gestational age (week)	
32–33	35 (3.4)
34–36	151 (14.9)
37–39	417 (41.0)
40–41	391 (38.5)
≥ 42	22 (2.2)
Birth weight (kg)	
0–1.3	3 (0.3)
1.4–2.2	61 (6.0)
2.3–3.1	343 (33.9)
≥ 3.2	604 (59.7)
Postnatal ICU admission	167 (16.4)
Previous hospital admission	162 (16.0)
Previous ICU admission	17 (1.7)
Previous breathing problems (count)	32 (3.2)
0	810 (79.7)
1	160 (15.7)
2	46 (4.5)
History of eczema	149 (14.7)
**Parent-reported symptoms at home**	
Poor feeding	32 (3.2)
Cyanosis within 24 h	92 (9.1)
Apnea	131 (12.9)
Apnea within 24 h	86 (8.5)
Duration of symptom ($\le$ 24 h)	53 (5.2)
**Signs and symptom at ED**	
Vital signs at presentation	
Temperature (F), median (IQR)	99.4 (98.8–100)
Pulse rate (bpm), median (IQR)	162 (150–176)
Respiratory rate (per min), median(IQR)	48 (40–60)
Use of supplemental oxygen (%)	51 (5)
Oxygen saturation level (%) at room air (IQR)	96 (94–98)
Oxygen saturation level (%) with the use of supplemental oxygen (IQR)	98 (95–100)
Wheeze	602 (62.3)
Severity of retraction	
None	192 (19.6)
Mild	431 (43.9)
Moderate/severe	358 (36.5)
Apnea	56 (5.5)
Dehydration	392 (39.5)
**Virology**	
RSV	821 (80.8)
**Length of hospital stay (days), range**	0–60
**Clinical outcomes**	
Positive pressure ventilation use^a^	55 (5.4)
Intensive treatment use^b^	163 (16.0)

Data are no. (%) of infants unless otherwise indicated. Percentages may not equal 100, because of rounding and missingness.

*bpm* beats per minute, *IQR* interquartile range, *ICU* intensive care unit, *RSV* respiratory syncytial virus.

^a^Infants with bronchiolitis who underwent continuous positive airway ventilation and/or mechanical ventilation.

^b^Infants with bronchiolitis who were admitted to ICU and/or who underwent positive pressure ventilation.

### Predicting positive pressure ventilation outcome

**Figure 1:**
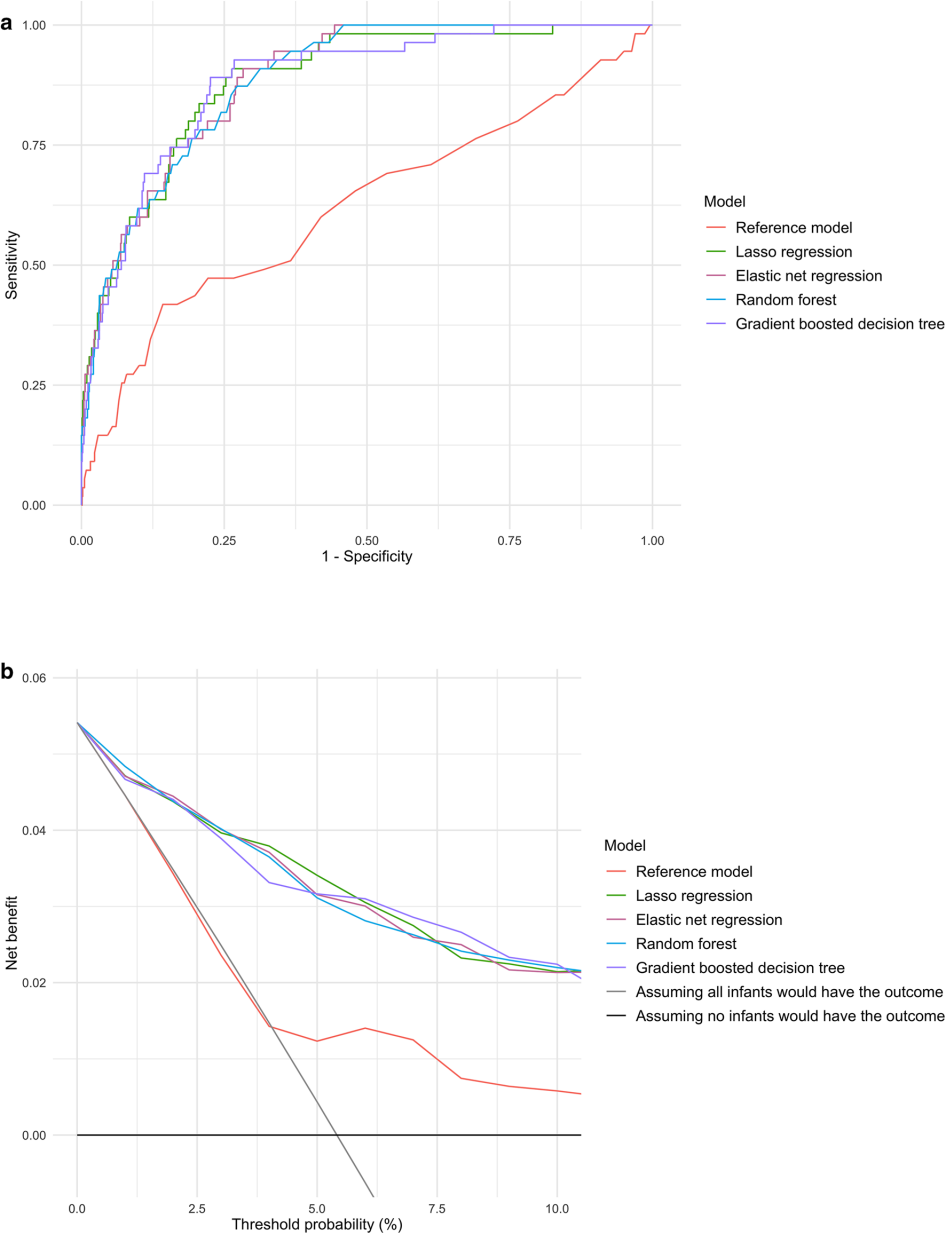
Prediction ability of the reference and machine learning models for positive pressure ventilation outcome in the overall cross-validation dataset. (**A**) Receiver-operating-characteristics (ROC) curves. The corresponding value of the area under the receiver-operating-characteristics curve (AUC) for each model are presented in Table 2. (**B**) Decision curve analysis. X-axis indicates the threshold probability for positive pressure ventilation outcome; Y-axis indicates the net benefit. Compared to the reference model, the net benefit of all machine learning models was larger over the range of clinical threshold.

In the prediction of positive pressure ventilation outcome, the discriminatory abilities of all models are summarized in Fig. 1A and Table 2. All four machine learning models demonstrated significantly superior AUCs (all P < 0.001). For example, compared with the reference model (AUC 0.62 [95% CI 0.53–0.70]), the AUC was higher in the elastic net regression (AUC 0.89 [95% CI 0.85–0.92]) and gradient boosted decision tree (AUC 0.88 [95% CI 0.84–0.93]) models. Similarly, compared with the reference model, all machine learning models also achieved a significant net reclassification improvement (all P < 0.001).

**Table 2:** Prediction performance of the reference, and machine learning models in infants hospitalized for bronchiolitis.

Outcomes and models	AUC	P-value^a^	NRI^b^	P-value^b^	Sensitivity	Specificity	PPV	NPV
**Positive pressure ventilation outcome**								
Reference model	0.62 (0.53–0.70)	Reference	Reference	Reference	0.62 (0.49–0.75)	0.57 (0.54–0.60)	0.075 (0.054–0.097)	0.96 (0.95–0.97)
Logistic regression with Lasso regularization	0.88 (0.84–0.93)	< 0.001	1.09 (0.87–1.32)	< 0.001	0.84 (0.73–0.93)	0.79 (0.77–0.82)	0.19 (0.14–0.24)	0.99 (0.99–0.99)
Logistic regression with elastic net regularization	0.89 (0.85–0.92)	< 0.001	1.05 (0.82–1.28)	< 0.001	0.89 (0.80–0.96)	0.73 (0.70–0.75)	0.15 (0.11–0.18)	0.99 (0.99–0.99)
Random forest	0.89 (0.85–0.92)	< 0.001	1.17 (0.96–1.38)	< 0.001	0.85 (0.75–0.95)	0.74 (0.71–0.76)	0.15 (0.12–0.21)	0.99 (0.99–0.99)
Gradient boosted decision tree	0.88 (0.84–0.93)	< 0.001	1.08 (0.84–1.33)	< 0.001	0.89 (0.80–0.96)	0.77 (0.75–0.80)	0.17 (0.08–0.21)	0.99 (0.99–0.99)
**Intensive treatment outcome**								
Reference model	0.62 (0.57–0.67)	Reference	Reference	Reference	0.58 (0.55–0.62)	0.58 (0.50–0.66)	0.21 (0.18–0.24)	0.88 (0.86–0.89)
Logistic regression with Lasso regularization	0.79 (0.76–0.83)	< 0.001	0.68 (0.52–0.84)	< 0.001	0.75 (0.69–0.82)	0.70 (0.66–0.73)	0.31 (0.26–0.38)	0.94 (0.93–0.94)
Logistic regression with elastic net regularization	0.80 (0.76–0.83)	< 0.001	0.58 (0.42–0.74)	< 0.001	0.72 (0.64–0.79)	0.74 (0.71–0.77)	0.33 (0.28–0.41)	0.93 (0.92–0.94)
Random forest	0.79 (0.75–0.84)	< 0.001	0.70 (0.55–0.86)	< 0.001	0.70 (0.63–0.77)	0.78 (0.76–0.81)	0.37 (0.29–0.45)	0.93 (0.92–0.94)
Gradient boosted decision tree	0.79 (0.75–0.84)	< 0.001	0.72 (0.57–0.87)	< 0.001	0.74 (0.67–0.80)	0.74 (0.71–0.77)	0.33 (0.26–0.42)	0.93 (0.92–0.94)

*AUC* area under the receiver-operating-characteristic curve, *NRI* net reclassification improvement, *PPV* positive predictive value, *NPV* negative predictive value.

^a^P-value was calculated to compare area-under-the-curve of the reference model with that of each machine model.

^b^We used continuous NRI and its P-value.

Additionally, compared with the reference model, all machine learning models also demonstrated a higher sensitivity (e.g., 0.62 [95% CI 0.49–0.75] in the reference model vs. 0.89 [95% CI 0.80–0.96] in the elastic net regression; Table 2) and specificity (e.g., 0.57 [95% CI 0.54–0.60] in the reference model vs. 0.79 [95% CI 0.77–0.82] in the Lasso regression model). More specifically, all machine learning models correctly predicted a larger number of infants who underwent positive pressure ventilation (true-positives) with a fewer number of predicted outcomes (Table 3). For example, the reference scoring system categorized most infants (n = 629, 62%) into the prediction score groups of 2–3. The reference model correctly identified 16 out of 25 infants who underwent positive pressure ventilation, while predicting that 265 infants would have undergone positive pressure ventilation. In contrast, the gradient boosted decision tree model correctly identified 23 (of 25) patients, while predicting that 135 infants would have undergone positive pressure ventilation in the same patient groups. Considering the low prevalence of the positive pressure ventilation outcome, all models had a high negative predictive value (e.g., 0.96 [95% CI 0.95–0.97] in the reference model vs. 0.99 [95% CI 0.99–0.99] in the Lasso regression model; Table 2).

**Table 3:** The number of actual and predicted outcomes of prediction models, according to the score of the reference model.

Reference model (score)	Positive pressure ventilation use n (%)	Reference model		Lasso regression		Elastic net regression		Random forest		Gradient boosted tree	
		Correctly identified outcome n (%)	Predicted outcome n	Correctly identified outcome n (%)	Predicted outcome n	Correctly identified outcome n (%)	Predicted outcome n	Correctly identified outcome n (%)	Predicted outcome n	Correctly identified outcome n (%)	Predicted outcome n
0: (n = 41)	1 (2.4)	0	6	0	7	0	8	1	9	0	8
1: (n = 64)	3 (4.7)	1	15	3	11	2	14	2	11	2	11
2: (n = 359)	13 (3.6)	9	156	12	80	11	106	11	78	12	79
3: (n = 270)	12 (4.4)	7	109	10	52	12	65	10	58	11	56
4: (n = 122)	3 (0.8)	2	46	1	20	1	30	2	41	1	24
5: (n = 58)	8 (13.8)	3	22	7	21	8	24	7	31	8	24
6: (n = 15)	0 (0.0)	0	3	0	6	0	6	0	8	0	6
7: (n = 41)	5 (12.5)	3	22	3	22	5	30	4	28	5	29
8: (n = 24)	4 (16.7)	2	8	4	12	4	14	4	17	4	15
9: (n = 11)	0 (0.0)	0	2	0	4	0	4	0	7	0	5
10: (n = 8)	5 (62.5)	3	3	5	7	5	7	5	8	5	7
11: (n = 3)	1 (33.3)	0	2	1	2	1	3	1	3	1	2
12: (n = 0)	0 (0)										
Overall (n = 1,016)	55 (5.4)	30 (55)	394	46 (84)	244	49 (89)	311	47 (85)	299	49 (89)	266

Reference model (Score)	Intensive treatment n (%)	Reference model		Lasso regression		Elastic net regression		Random forest		Gradient boosted tree	
		Correctly identified outcome n (%)	Predicted outcome n	Correctly identified outcome n (%)	Predicted outcome n	Correctly identified outcome n (%)	Predicted outcome n	Correctly identified outcome n (%)	Predicted outcome n	Correctly identified outcome n (%)	Predicted outcome n
0: (n = 41)	2 (4.9)	1	10	1	8	0	5	0	4	0	4
1: (n = 64)	8 (12.5)	1	11	5	17	5	11	5	12	5	9
2: (n = 359)	44 (12.2)	21	157	27	133	23	111	26	77	24	87
3: (n = 270)	36 (13.3)	18	118	28	75	28	71	23	64	28	75
4: (n = 122)	17 (13.8)	8	53	9	36	9	33	7	40	10	53
5: (n = 58)	17 (29.3)	6	26	14	30	14	30	15	28	15	29
6: (n = 15)	3 (20.0)	0	3	3	7	2	7	3	9	3	12
7: (n = 41)	19 (47.5)	15	28	19	38	19	36	18	32	18	35
8: (n = 24)	7 (29.2)	4	8	7	18	7	17	7	16	7	17
9: (n = 11)	1 (9.1)	0	3	1	10	1	9	1	6	1	7
10: (n = 8)	7 (87.5)	3	4	7	7	7	7	7	8	7	8
11: (n = 3)	2 (66.7)	0	1	2	3	2	3	2	3	2	3
12: (n = 0)	0 (0)										
Overall (n = 1,016)	163 (16.0)	77 (47)	422	123 (75)	382	117 (72)	340	114 (70)	299	120 (74)	339

Likewise, in the decision curve analysis (Fig. 1B), all four machine learning models outperformed the reference model, demonstrating a greater net benefit throughout the range of clinical thresholds, indicating that the machine learning prediction would more accurately identify high-risk infants (true-positives) while taking the trade-off with false-positives into consideration.

### Predicting intensive treatment outcome

**Figure 2:**
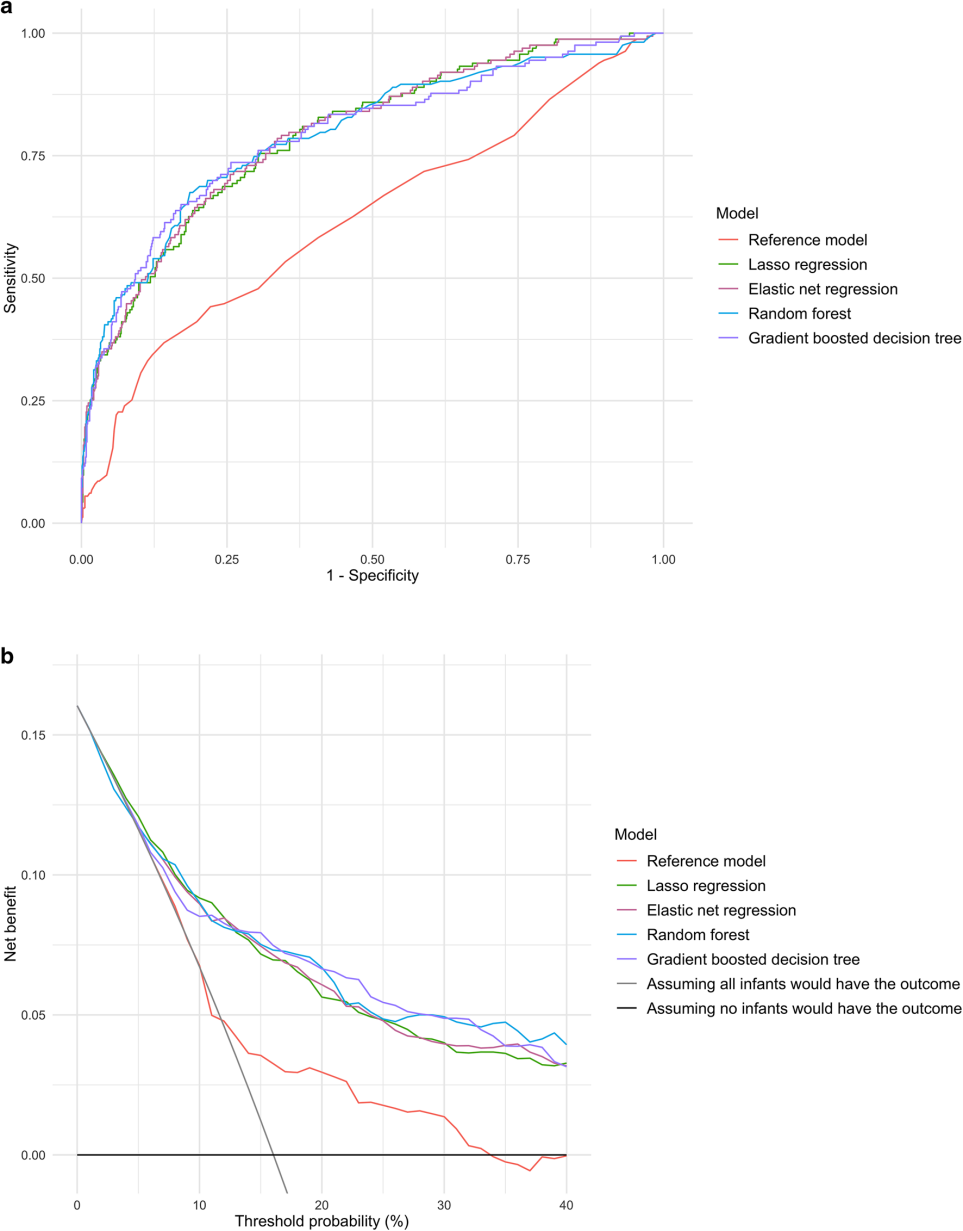
Prediction ability of the reference and machine learning models for intensive treatment outcome in the overall cross-validated dataset. (**A**) Receiver-operating-characteristics (ROC) curves. The corresponding values of the area under the receiver-operating-characteristics curve (AUC) for each model are presented in Table 2. (**B**) Decision curve analysis. X-axis indicates the threshold probability for intensive treatment outcome; Y-axis indicates the net benefit. Compared to the reference model, the net benefit of all machine learning models was larger over the range of clinical threshold.

In the prediction of intensive treatment outcome, the discriminatory abilities of all models are shown in Fig. 2A and Table 2. All four machine learning models demonstrated a significantly higher AUC (all P < 0.001). For example, compared with the reference model (AUC 0.62 [95% CI 0.57–0.67]), the AUC was higher in the elastic net regression (AUC 0.80 [95% CI 0.76–0.83]) and random forest (AUC 0.79 [95% CI 0.75–0.84]) models. Similarly, compared with the reference model, all machine learning models also achieved significant net reclassification improvement (all P < 0.001).

Additionally, all machine learning models demonstrated a higher sensitivity (e.g., 0.58 [95% CI 0.49–0.75] in the reference model vs. 0.75 [95% CI 0.69–0.82] in the Lasso regression; Table 2) and specificity (e.g., 0.58 [95% CI 0.50–0.66] in the reference model vs. 0.78 [95% CI 0.76–0.81] in the random forest model). For example, among the infants categorized into the reference score groups of 2–3 (62% of cohort infants), the reference model correctly identified 39 out of 80 infants who had intensive treatment, while predicting that 275 infants would have had intensive treatment (Table 3). In contrast, the gradient boosted decision tree correctly identified 52 (out of 80) infants with the outcome, while predicting that 162 infants would have had intensive treatment. Likewise, in the decision curve analysis (Fig. 2B), all four machine learning models outperformed the reference model, demonstrating a greater net benefit throughout the range of clinical thresholds.

### Variable importance

To yield insights into the relevance of each predictor, eFigures 1 and 2 (Additional file 1) summarized the 15 most important predictors of random forest and gradient boosted decision tree models for each outcome. In the prediction of positive pressure ventilation outcome, age, oxygen saturation level with the use of supplemental oxygen, and other vital signs [at the presentation] were the most important predictors in both models (eFigures 1A and 2A). Likewise, in the prediction of intensive treatment outcome, similar predictors were considered important in the both models (eFigures 1B and 2B).

## Discussion

In this analysis of multicenter prospective cohort data from 1,016 infants, we applied four modern machine learning approaches (i.e., Lasso regression, elastic net regression, random forest, and gradient boosted decision tree) to the prediction of acute severity outcomes of bronchiolitis. Compared to the reference model that was derived in an ED sample^6^, these machine learning models consistently demonstrated a superior performance in predicting positive pressure ventilation and intensive treatment outcomes, including AUC and net reclassification. Additionally, the machine learning models achieved a higher sensitivity and specificity for the two outcomes, in both the overall cohort and the majority of cohort infants that were categorized into the reference score groups of 2–3. Furthermore, the decision curve analysis also demonstrated the net benefit of machine learning models was also greater—i.e., a larger number of true-positives considering a trade-off with false-positives—across a range of clinical thresholds. To the best of our knowledge, this is the first study that has investigated the performance of modern machine learning models in predicting severity in infants with bronchiolitis.

One of the main objectives in the risk stratification of infants with bronchiolitis is to promptly identify infants at risk for higher severity and efficiently utilize finite healthcare resources. The American Academy of Pediatrics bronchiolitis guideline^2^ highlights the importance of assessing the risk in infants with bronchiolitis. However, optimal risk stratification and prediction remains a challenge as the clinical course in this population (even in infants hospitalized for bronchiolitis) is highly variable^10–12^. Previous studies, by using conventional modeling (e.g., logistic regression models), have reported a moderate ability to predict severity outcomes (e.g., ED-to-hospital admission, hospital length-of-stay, ICU admission, positive pressure ventilation use) of infants with bronchiolitis^6–9,19^. Although the use of an expanded set of predictors—e.g., repeated examinations and invasive monitoring during hospital course—may yield better predictive performance, it is often impractical in the real-world acute care settings with an aim to promptly risk-stratify these infants. Alternatively, the use of advanced machine learning models may improve the clinician’s decision-making ability. Indeed, machine learning models have recently been applied to the prediction of various disease conditions and clinical settings, such as early identification of mortality risk in patients with sepsis^13^, rehospitalization in patients with heart failure^20^, intensive treatment outcomes in patients with asthma exacerbation^15^, unplanned transfer to ICU^18^, and escalated care at pediatric ED triage^16^. Our multicenter study builds on these earlier reports, and extends them by demonstrating that the modern machine learning models outperform conventional approaches in predicting higher severity of infants with bronchiolitis. While external validation is warranted, these machine learning models using routinely-available predictors can be implemented to clinical practice (e.g., online risk calculators or build-in risk assessment systems)—similar to existent clinical scoring rules.

Clinical prediction systems strive for an appropriate balance between sensitivity and specificity because of the trade-off relationship between these two factors in the context of prevalence of clinical outcomes. In the present study, we observed that the reference score model did not effectively categorize most infants (i.e., 62% of cohort were categorized into the two score groups) or appropriately predicted infants who developed the outcomes. By contrast, the machine learning models correctly identified a larger number of true-positives (i.e., higher sensitivity). This finding supports the utility of these models in the target population, for which the one of the major priorities is to reduce “missed” high-risk cases. Additionally, the machine learning models also had a fewer number of false-positives (i.e., higher specificity) in predicting both outcomes while they were imperfect in the setting of relatively-smaller prevalence of outcome (5.4% for positive pressure ventilation use). This may mitigate excessive resource use in this large population. These findings are further supported by the decision curve analysis that demonstrated a greater net benefit of the machine learning models incorporating the trade-offs between true-positives and false-positives across the wide ranges of clinical thresholds.

There are several potential explanations for the observed gains in the predictive abilities of machine learning models. For example, machine learning models incorporate high-order interactions between predictors and nonlinear relationships with outcomes. Additionally, machine learning models are able to mitigate potential overfitting by adopting several methods, such as regularization, out-of-bagging estimation, and cross-validation. Furthermore, the use of large multicenter data with rigorous quality assurance might have contributed to low bias and variance in the machine models. Although the machine learning models achieved superior predictive ability, their performance remained imperfect. This may be explained, at least partially, by the limited set of predictors, subjectivity of some data elements (e.g., parent-reported symptoms at home), variable clinical factors after prehospitalization assessment (e.g., ED management and patient responses), difference in clinician’s practice patterns, and availability of intensive care resources. Notwithstanding the complexity and challenges of clinical prediction in infants with bronchiolitis, machine learning models have scalable advantages in the era of health information technology, such as automated sophistication of models through the sequential extraction of electronic health records, continuous non-invasive physiological monitoring, natural language processing, and reinforcement learning^21–24^. In the past, this scalability had not been attainable with the use of conventional approaches. Taken together, our findings and recent developments support cautious optimism that modern machine learning may enhance the clinician’s ability as an assistive technology.

Our study has several potential limitations. Firstly, the data may be subject to measurement bias and missingness. However, the study was conducted by trained investigators using a standardized protocol, which led to the low proportion of missingness in the predictors (eTable 3 in Additional file 1). Secondly, the clinical thresholds for these outcomes may depend on local resources and vary between clinicians and hospitals (e.g., different criteria for admission to the ICU). Yet, the decision curve analysis demonstrated the greater benefit of the machine learning models across the wide range of clinical thresholds. Lastly, the study cohort consisted of a racially/ethnically- and geographically-diverse US sample of infants hospitalized with bronchiolitis. While the severity of this population was highly variable and the model used pre-hospitalization data, our models might not be generalizable to infants in ambulatory settings. External validation of the models in different populations and settings is necessary. Nonetheless, our data remain highly relevant for the 107,000 infants hospitalized yearly in the US^1^.

## Conclusion

Based on data from a multicenter prospective cohort of 1,016 infants with bronchiolitis, we developed four machine learning models to predict severity of illness. By using prehospitalization data as predictors, these models consistently yielded superior performance—a higher AUC, net reclassification, sensitivity, and specificity—in predicting positive pressure ventilation and intensive treatment outcomes over the reference model^6^. Specifically, these advanced machine learning models correctly predicted a larger number of infants with higher severity—with a fewer number of false-positives—who would not be appropriately predicted by the conventional models. Moreover, the machine learning models also achieved a greater net benefit across wide ranges of clinical thresholds. Although an external validation is warranted, the current study lends support to the application of machine learning models to the prediction of acute severity in infants with bronchiolitis. Machine learning models have a potential to enhance clinicians’ decision-making ability and hence to improve clinical care and optimize resource utilization in this high morbidity population.

## Methods

### Study design, setting and participants

The current study aimed to develop machine learning models that accurately predict acute severity in infants with bronchiolitis, by using the data from a multicenter prospective cohort study of 1,016 infants hospitalized for bronchiolitis—the 35th Multicenter Airway Research Collaboration (MARC-35) study^25,26^. MARC-35 is coordinated by the Emergency Medicine Network (EMNet, https://www.emnet-usa.org^27^) an international research collaboration with 246 participating hospitals. Briefly, at 17 sites across 14 U.S. states (eTable 1 in Additional file 1), MARC-35 enrolled infants (aged < 1 year) who were hospitalized with an attending physician diagnosis of bronchiolitis during three consecutive bronchiolitis seasons (November 1 to April 30) during 2011–2014. The diagnosis of bronchiolitis was made according to the American Academy of Pediatrics bronchiolitis guidelines^2^, defined as acute respiratory illness with a combination of rhinitis, cough, tachypnea, wheezing, crackles, and retractions. We excluded infants who were transferred to a participating hospital > 24 h after initial hospitalization or with a preexisting heart and lung disease, immunodeficiency, immunosuppression or gestational age of < 32 weeks.

We followed the Standards for Reporting Diagnostic Accuracy statement guideline for the reporting of prediction models^28^. The institutional review board of the 17 participating hospitals (Alfred I. duPont Hospital for Children, Arnold Palmer Hospital for Children, Boston Children's Hospital, Children's Hospital of Los Angeles, Children's Hospital of Philadelphia, Children's Hospital of Pittsburgh, The Children's Hospital at St. Francis, The Children's Mercy Hospital & Clinics, Children's National Medical Center, Cincinnati Children's Hospital and Medical Center, Connecticut Children's Medical Center, Dell Children's Medical Center of Central Texas, Norton Children's Hospital, Massachusetts General Hospital, Phoenix Children's Hospital, Seattle Children's Hospital, Texas Children's Hospital) approved the study. Written informed consent was obtained from the parent or guardian.

### Predictors

For predictors in the machine learning models, we selected variables based on clinical plausibility and a priori knowledge^3,6–9,29–31^. These predictors—which are available in most prehospitalization settings—included demographics (age, sex, and race/ethnicity), medical history (prenatal maternal smoking, gestational age, birth weight, postnatal ICU admission, history of hospital and ICU admission, history of breathing problems, and history of eczema), parent-reporting symptoms (poor feeding, cyanosis, apnea, and duration of symptoms), ED presentation (vital signs [temperature, pulse rate, respiratory rate, oxygen saturation], interaction between oxygen saturation and supplemental oxygen use, wheezing, retractions, apnea, and dehydration), and detection of respiratory syncytial virus (RSV) by PCR^25^. These clinical data were obtained through a structured interview and medical record review by trained physicians and investigators using a standardized protocol^26^. All data were reviewed at the EMNet Coordinating Center at Massachusetts General Hospital (Boston, MA), and site investigators were queried about missing data and discrepancies identified by manual data checks.

### Outcomes

The primary outcome was the use of positive pressure ventilation—continuous positive airway pressure ventilation and/or intubation during inpatient stay^32^. The secondary outcome was intensive treatment defined as a composite of ICU admission and/or the use of positive pressure ventilation during the inpatient stay^3, 31^. In this observational study, patients were managed at the discretion of treating physicians. These two outcomes have been employed for outcomes in the MARC-35 study.

### Statistical analysis

In the training sets (80% randomly-selected samples) in fivefold cross-validation, we developed five models: the reference model^6^ and four machine learning models for each outcome. As the reference model, we fit logistic regression models using the predictors of a previously-established clinical prediction score that was derived using an ED sample^6^. We selected this prediction score as the reference model since it was recently developed in a large sample and focused on similar clinical outcomes reflecting acute severity of bronchiolitis^6,33^. The predictors included age, poor feeding, oxygen saturation, retractions, apnea, and dehydration, excluding nasal flaring/grunting, based on the availability of data in the current study (eTable 2).

Next, using the prehospitalization predictors, we developed four machine learning models: (1) logistic regression with Lasso regularization (Lasso regression), (2) logistic regression with elastic net regularization (elastic net regression), (3) random forest, and (4) gradient boosted decision tree models. First, Lasso regression is an extension of regression-based models that has an ability to shrink (or regularize) the predictor coefficients toward zero, thereby effectively selecting important predictors and improving interpretability of the model^34^. Lasso regression computes the optimal regularization parameter (lambda) that minimizes the sum of least square plus L1-shrinkage penalty using a cross-validation method^35^. Second, elastic net regression is another regression-based model incorporating both Lasso-regularization and Ridge-regularization^34,36^. Elastic net regression calculates the optimal regularization parameter that minimizes the sum of least square plus weighted L1-shrinkage penalty and weighted L2-shrinkage penalty. We used R *glmnet* and *caret* packages for Lasso regression and elastic net regression models^37,38^. Third, random forest is an ensemble of decision trees generated by bootstrapped training samples with random predictor selection in tree induction^34,39^. We created a hyperparameter tuning grid to identify the best set of parameters using cross-validation methods. We used *randomForest* and *caret* packages to construct random forest models^38,40^. Lastly, gradient boosted decision tree is another ensemble method which constructs new simple tree models predicting the errors and residuals of the previous model. When adding a new tree, this model uses a gradient descent algorithm minimizes a loss function^41^. We performed hyperparameter tuning sequentially using a fivefold cross-validation method. We used R *xgboost* and *caret* packages to construct gradient boosted decision tree^38,42^. To minimize potential overfitting, we utilized several methods—e.g., regularizations (or penalizations) in Lasso and elastic net regression models, out-of-bag estimation in random forest models, and cross-validation in all models.

As for the predictor engineering methods of the machine learning models, we preprocessed predictors sequentially. First, we investigated non-linear relationships between the continuous predictors and outcomes and created quadric terms of age, respiratory rate, and temperature. These quadratic terms were used only for regression-based machine learning models (i.e., logistic regression models with Lasso regularization and those with elastic net regularization). Second, we also chose either of highly-correlated predictors (e.g., age and weight at hospitalization). Third, we imputed predictors with missing values (eTable 3) using bagged tree imputation. Fourth, we converted continuous predictors into normalized scales using Yeo-Johnson transformation. Categorical predictors were coded as dummy variables while birth weight, gestational age, previous breathing problem, and degree of retraction were coded as ordinal variables. Fifth, to incorporate clinically evident interaction between oxygen saturation level and use of supplemental oxygen, we created an interaction term between oxygen saturation and use of supplemental oxygen. Lastly, we removed predictors that are highly sparse in the dataset. We applied these preprocessing methods independently to the training sets and the test sets to avoid carrying the information from the training sets to the test sets. We used R *recipe* package for these predictor preprocessing^43^.

To examine the variable importance in the random forest, we used permutation-based variable importance—normalized average values of difference between the prediction accuracy of out-of-bag estimation and that of the same measure after permutating each predictor. In the gradient boosted model, we also computed the variable importance that is summed over iterations^39^. We graphically presented the rank of variable importance using unscaled values.

To measure the test performance of each model, we computed the overall cross-validation performance from the test sets (the remaining randomly-selected 20% samples). As the predictive performance, we used (1) the area under the receiver-operating-characteristic curve (AUC), (2) net reclassification improvement, (3) confusion matrix results (i.e., sensitivity, specificity, positive predictive value, and negative predictive value), and (4) net benefit from decision curve analysis. To compare the AUC between the models, we used Delong’s test^44^. To compute AUC and its confidential interval, we used *pROC* package^45^. We also used the net reclassification improvement to quantify whether a new model provides clinically relevant improvements in prediction when compared to the reference model^46^. To compute the net reclassification improvement, we used *PredictABEL* package^47^. To address the class imbalance in the both outcomes, we employed the value with the shortest distance to the top-left part of the AUC plot as the threshold for the confusion matrix^39^.The decision curve analysis incorporates the information on both the benefit of correctly predicting the outcome (true-positives) and the relative harm of incorrectly labelling patients as if they would have the outcome (false-positives)—i.e., the net benefit^48–52^. We made a graphical presentation of the net benefit for each model over a range of threshold probabilities (or clinical preferences) of the outcome as decision curves. We used decision curve analysis R source code from Memorial Sloan Kettering Cancer Center^53^ and plotted the graphs using *ggplot2* package^54^. We performed all analysis with R version 3.5.1 (R Foundation for Statistical Computing, Vienna, Austria)^55^.

## Supplementary information

Supplementary file1 (DOCX 21137 kb)

## Data Availability

The datasets generated and analysed during the current study are not publicly available because of the informed consent documents. Per the informed consent documents of the MARC research participants, the data sharing and use are limited to the severe bronchiolitis, recurrent wheezing, asthma and related concepts. Accordingly, the data are not publicly available but available from the corresponding author on reasonable request.

## Abbreviations

MARC-35: The 35th Multicenter Airway Research Collaboration study
AUC: Area-under-the-curve
CI: Confidential interval
ICU: Intensive care unit
ED: Emergency department
EMNet: Emergency Medicine Network
RSV: Respiratory syncytial virus
IQR: Interquartile range
NRI: Net reclassification improvement
PPV: Positive predictive value
NPV: Negative predictive value
