# Supplementary material for: Machine learning-based prediction of acute severity in infants hospitalized for bronchiolitis: a multicenter prospective study
Source: Sci Rep. 2020 Jul 3;10:10979. doi: 10.1038/s41598-020-67629-8 (PMC7335203; doi:10.1038/s41598-020-67629-8)
Supplement: Supplementary file 1 — Supplementary file1 (DOCX 21137 kb) [file 41598_2020_67629_MOESM1_ESM.docx]

**ADDITIONAL FILE 1**

**Machine learning-based prediction of acute severity in infants hospitalized for bronchiolitis: A multicenter prospective study**

**Authors**: Yoshihiko Raita, MD, MPH^1^; Carlos A. Camargo, Jr. MD, DrPH^1^; Charles G. Macias,

MD, MPH^2^; Jonathan M. Mansbach, MD, MPH^3^; Pedro A. Piedra, MD^4^; Stephen C. Porter, MD, MPH, MSc^5,6^; Stephen J. Teach, MD, MPH^7^; and Kohei Hasegawa, MD, MPH^1^

**Affiliations**:

1. Department of Emergency Medicine, Massachusetts General Hospital, Harvard Medical School, Boston, MA, USA
2. Department of Pediatric Emergency Medicine, Rainbow Babies and Children’s Hospital, OH, USA
3. Department of Medicine, Boston Children's Hospital, Harvard Medical School, Boston,

MA, USA

1. Departments of Molecular Virology and Microbiology and Pediatrics, Baylor College of

Medicine, Houston, TX, USA

1. Department of Pediatrics, University of Cincinnati, College of Medicine, Cincinnati, OH
2. Division of Emergency Medicine, Cincinnati Children’s Hospital Medical Center, Cincinnati, OH
3. Division of Emergency Medicine and Department of Pediatrics, Children’s National

Health System, Washington, DC, USA

**Contents**

**eTable 1.** Principal investigators at the 17 participating sites in MARC-35

**eTable 2.** Predictors and scores of the reference model in 1,016 infants hospitalized for bronchiolitis

**eTable 3.** Proportion of missing predictors in 1,016 infants hospitalized for bronchiolitis

**eFigure 1.** Variable importance plot of random forest model

**eFigure 2.** Variable importance plot of gradient boosted decision tree model

**eTable 1.** **Principal investigators at the 17 participating sites in MARC-35**

| Amy D. Thompson, MD | Alfred I. duPont Hospital for Children, Wilmington, DE |
| --- | --- |
| Federico R. Laham, MD, MS | Arnold Palmer Hospital for Children, Orlando, FL |
| Jonathan M. Mansbach, MD, MPH | Boston Children's Hospital, Boston, MA |
| Vincent J. Wang, MD, MHA and Susan  Wu, MD | Children's Hospital of Los Angeles, Los Angeles, CA |
| Michelle B. Dunn, MD and Jonathan M.  Spergel, MD, PhD | Children's Hospital of Philadelphia, Philadelphia, PA |
| Juan C. Celedón, MD, DrPH | Children's Hospital of Pittsburgh, Pittsburgh, PA |
| Michael R. Gomez, MD, MS-HCA and  Nancy Inhofe, MD | The Children's Hospital at St. Francis, Tulsa, OK |
| Brian M. Pate, MD and Henry T. Puls, MD | The Children's Mercy Hospital & Clinics, Kansas City, MO |
| Stephen J. Teach, MD, MPH | Children's National Medical Center, Washington, D.C. |
| Richard T. Strait, MD and Stephen C.  Porter, MD, MSc, MPH | Cincinnati Children's Hospital and Medical Center, Cincinnati, OH |
| Ilana Y. Waynik, MD | Connecticut Children's Medical Center, Hartford, CT |
| Sujit Iyer, MD | Dell Children's Medical Center of Central Texas, Austin, TX |
| Michelle D. Stevenson, MD, MS | Norton Children's Hospital, Louisville, KY |
| Wayne G. Shreffler, MD, PhD and Ari R.  Cohen, MD | Massachusetts General Hospital, Boston, MA |
| Anne K. Beasley, MD and Cindy S. Bauer,  MD | Phoenix Children's Hospital, Phoenix, AZ |
| Thida Ong, MD and Markus Boos, MD,  PhD | Seattle Children's Hospital, Seattle, WA |
| Charles G. Macias, MD, MPH | Texas Children's Hospital, Houston, TX |

**eTable 2. Predictors and scores of the reference models in 1,016 infants hospitalized for bronchiolitis**

| **Scores and predictors** | | **Overall**  n=1,016 | |
| --- | --- | --- | --- |
| Reference model^a^ | Overall score, median(IQR) | 3 | (2-4) |
|  | Age ≤2 month | 311 | (30.6) |
|  | Poor feeding (reported on history) | 32 | (3.2) |
|  | Oxygen saturation <90% | 85 | (9.0) |
|  | Apnea (history or observed in the ED) | 153 | (15.1) |
|  | Dehydration (observed in ED) | 392 | (39.5) |
|  | Retraction | 789 | (80.4) |
| Abbreviations: ED, emergency department; IQR, interquartile range | | | |
| Data are no. (%) of infants unless otherwise indicated. Percentages may not equal 100, because of rounding and missingness. | | | |
| ^a^ Original model developed by *Freire et al.*^1^ includes nasal flaring and/or grunting variable that is not available in the MARC-35 data. | | | |

**eTable 3.** **Proportion of missing data in 1,016 infants hospitalized for bronchiolitis**

| **Variables** | **Missing**  **n (%)** | |
| --- | --- | --- |
|  |  |  |
| Demographics: |  |  |
| Age | 0 | (0) |
| Female sex | 0 | (0) |
| Race/ethnicity | 0 | (0) |
| Medical history: |  |  |
| Prenatal maternal smoking | 18 | (1.8) |
| Gestational age | 0 | (0) |
| Birth weight | 5 | (0.5) |
| Postnatal ICU admission | 0 | (0) |
| Previous hospital admission | 2 | (0.2) |
| Previous ICU admission | 0 | (0) |
| Previous breathing problems | 0 | (0) |
| History of eczema | 1 | (0.1) |
| Parent-reporting symptoms at home |  |  |
| Poor feeding | 1 | (0.1) |
| Cyanosis within 24 hours | 0 | (0) |
| Apnea | 0 | (0) |
| Apnea within 24 hours | 0 | (0) |
| Duration of symptom | 0 | (0) |
| Signs and symptom at ED |  |  |
| Vital signs at presentation |  |  |
| Temperature | 17 | (1.7) |
| Pulse rate | 19 | (1.9) |
| Respiratory rate | 19 | (1.9) |
| Use of supplemental oxygen | 16 | (1.6) |
| Oxygen saturation | 21 | (2.1) |
| Oxygen saturation with use of supplemental oxygen | 1 | (0.1) |
| Wheeze | 49 | (4.8) |
| Severity of retraction | 35 | (3.4) |
| Apnea | 0 | (0) |
| Dehydration | 23 | (2.3) |
| Virology | 0 | (0) |
| Clinical outcomes |  |  |
| Positive pressure ventilation use^a^ | 0 | (0) |
| Intensive treatment use^b^ | 0 | (0) |
| Abbreviations: ED, emergency department; ICU, intensive care unit; RSV, respiratory syncytial virus |  |  |
| ^a^ Infants with bronchiolitis who underwent continuous positive airway ventilation and/or mechanical ventilation. | | |
| ^b^ Infants with bronchiolitis who were admitted to ICU and/or who underwent positive pressure ventilation. | | |

**eFigure 1. Variable importance plot of random forest model**

**A)** Positive pressure ventilation outcome


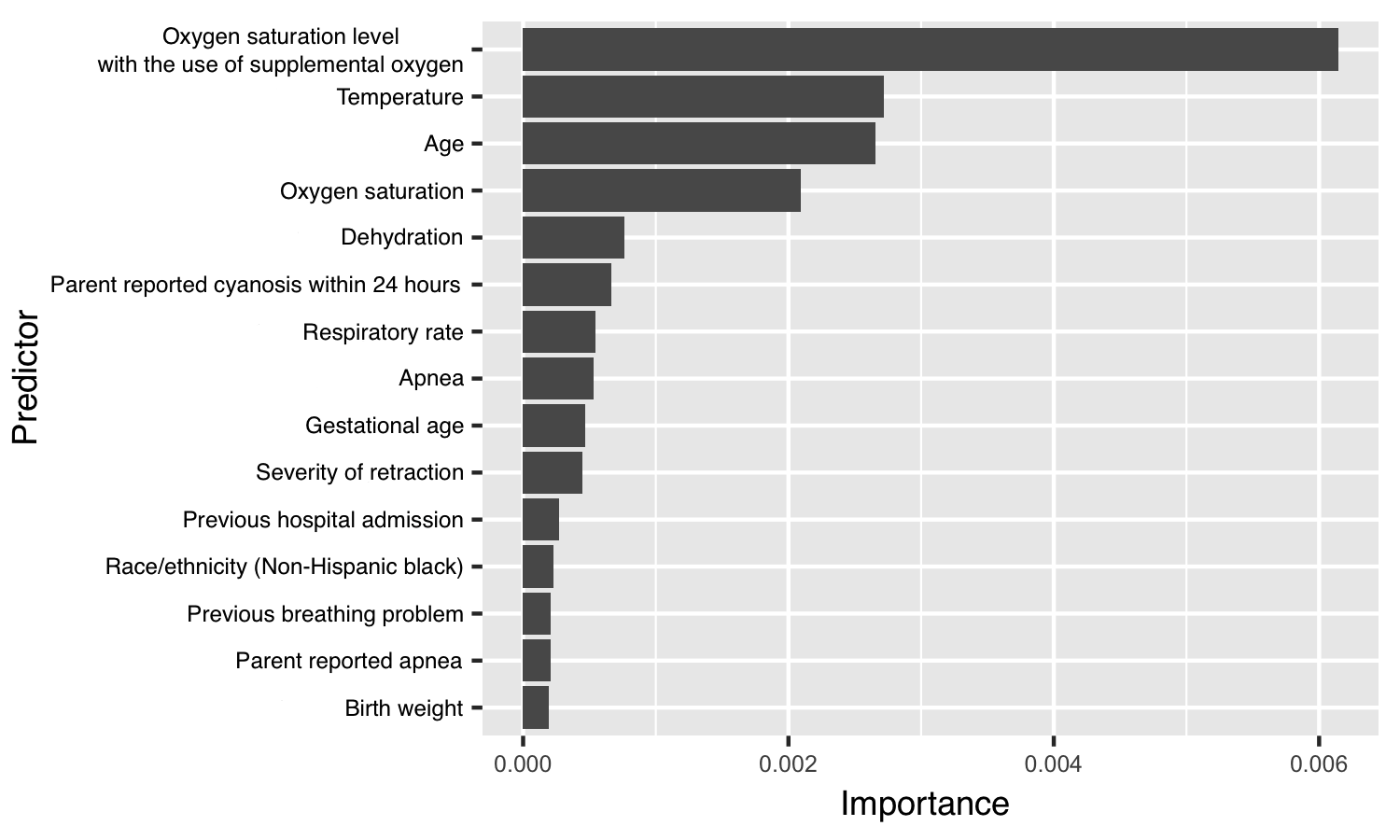


**B)** Intensive treatment outcome

**
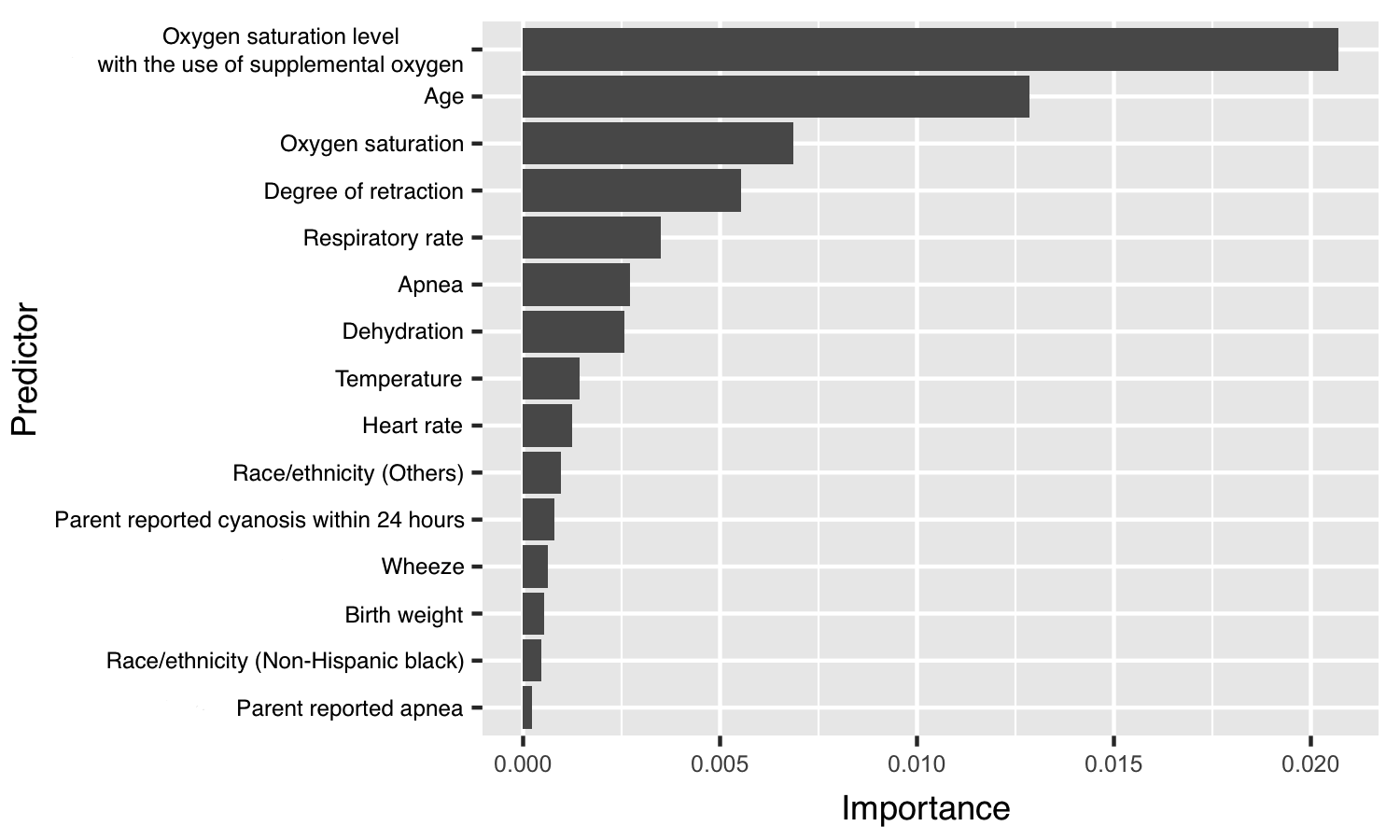
**

**eFigure 2. Variable importance plot of gradient boosted decision tree model**

**A)** Positive pressure ventilation outcome

**
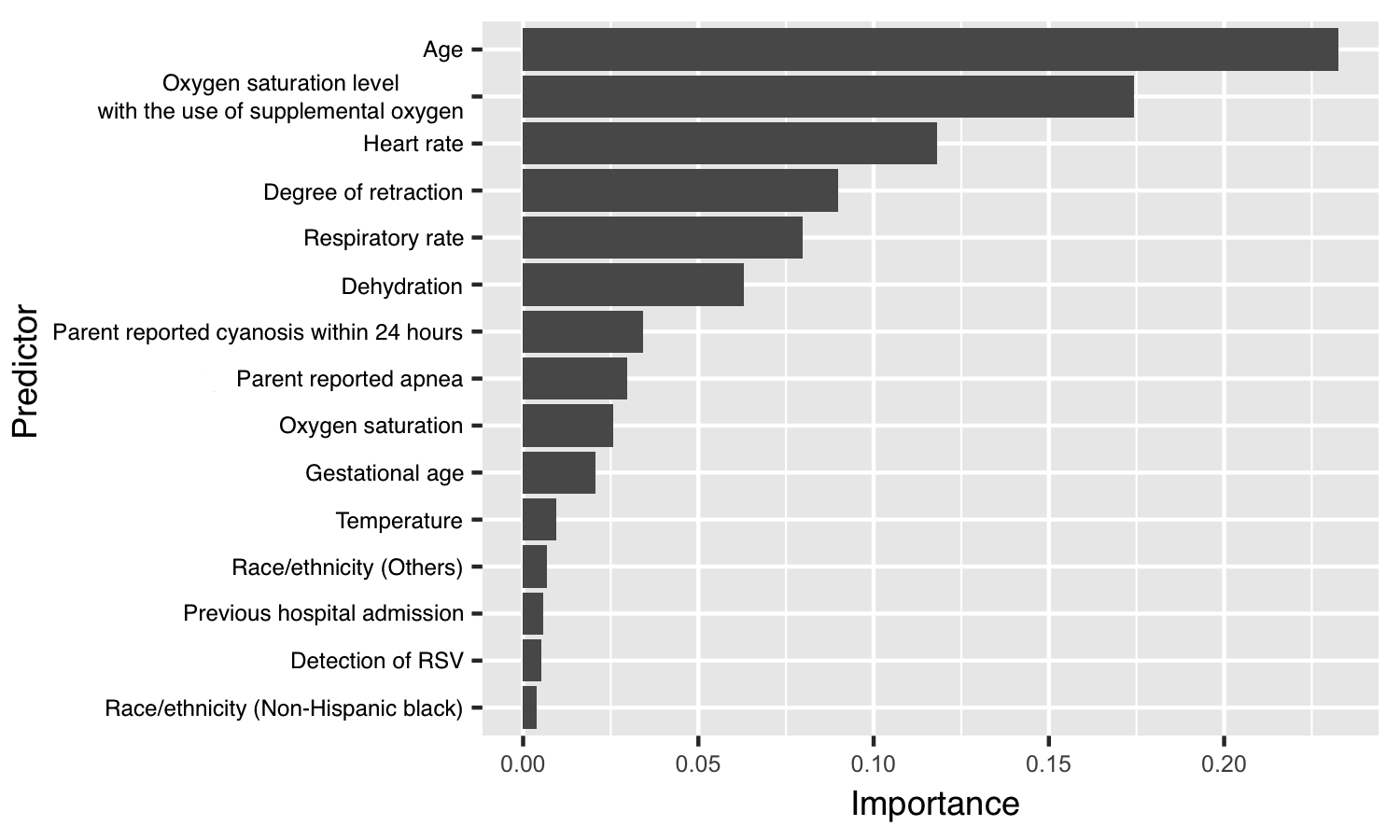
**

**B)** Intensive treatment outcome

**
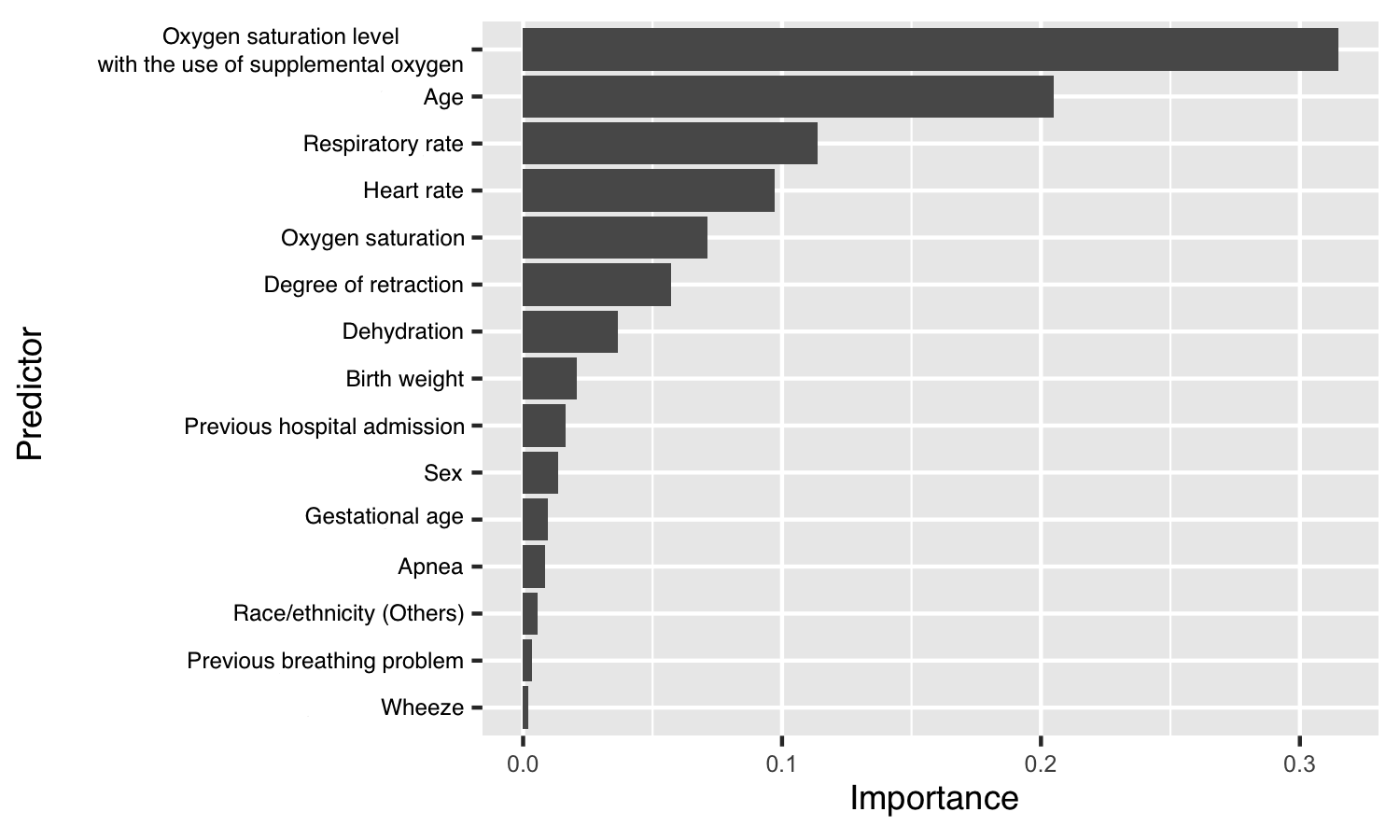
**
